# Molecular Identification and Selection of Probiotic Strains Able to Reduce the Serum TMAO Level in Mice Challenged with Choline

**DOI:** 10.3390/foods10122931

**Published:** 2021-11-27

**Authors:** Latha Ramireddy, Hau-Yang Tsen, Yu-Chen Chiang, Chen-Ying Hung, Shih-Rong Wu, San-Land Young, Jin-Seng Lin, Chien-Hsun Huang, Shih-Hau Chiu, Chien-Chi Chen, Chih-Chieh Chen

**Affiliations:** 1 Department of Food Science and Technology, Hung Kuang University, No. 1018, Sec. 6, Taiwan Boulevard, Shalu District, Taichung 43302, Taiwan; ps20874ss@gmail.com; 2 Department of Food Nutrition and Biotechnology, Asia University, Taichung 41354, Taiwan; honda224@asia.edu.tw; 3 Department of Internal Medicine, Taipei Veterans General Hospital, Hsinchu Branch, Hsinchu 310, Taiwan; kndshung@gmail.com; 4 Culture Collection and Research Institute, Synbio Tech Inc., Kaohsiung 82151, Taiwan; s333@synbiotech.com.tw (S.-L.Y.); jslin@synbiotech.com.tw (J.-S.L.); 5 Bioresource Collection and Research Center, Food Industry Research and Development Institute, 331 Shih-Pin Rd, Hsinchu 30062, Taiwan; chh@firdi.org.tw (C.-H.H.); shc@firdi.org.tw (S.-H.C.); chj@firdi.org.tw (C.-C.C.); 6 Institute of Medical Science and Technology, National Sun Yat-sen University, Kaohsiung 80424, Taiwan; chieh@imst.nsysu.edu.tw

**Keywords:** TMAO, cardiovascular disease, choline-fed mice, lactobacilli, *Lpb. plantarum*, *L. amylovorus*, WgMLST

## Abstract

Trimethylamine oxide (TMAO) originates from trimethylamine (TMA), which is oxidized in the liver by hepatic flavin-containing monooxygenases (FMO3). TMA is produced by its dietary precursors such as choline, carnitine, and phosphatidylcholine by gut microbiota. TMAO attracts attention, identified as a novel and independent risk factor for promoting obesity, atherosclerosis and cardiovascular disease (CVD), chronic kidney disease (CKD), insulin tolerance, and colon cancer. Probiotics have been considered as live microorganisms, providing benefits to their host when they are given in sufficient quantities and administered continuously. The objective of this study is to suggest a method to select potential probiotic strains to reduce the serum concentration of TMAO in mice fed with choline. In this work, we chose three lactobacilli with strong adherence capability, and fed multistrain formula (MF) to the mice challenged with choline. On days 7, 14, and day 28, it was found that the MF-containing *L. amylovorus* LAM1345, *Lpb. plantarum* LP1145, and *Lim. fermentum* LF33 showed a significant reduction in serum TMAO and TMA levels. For the single strains, LP1145 reduced TMAO on days 14 and 28, and strain LAM1345 reduced TMAO significantly on days 7 and day 14. For strain LF1143 from strain LF33, it showed no significant effect on TMAO and TMA. Thus, MF showed the best effect, which may be due to the additive and synergetic effect and the contribution of strain LP1145 and LAM1345. Finally, for the LAM1345 and LP1145 strains, we used molecular identification and typing methods to assure that these two strains are unique strains. The methods used for LAM 1345 were leader peptidase A (lepA) gene analysis and phylogenetic analysis, while for strain LP 1145and other strains of *Lpb. plantarum* subsp. *plantarum* sequences were compared using the whole-genome multilocus sequence typing (wgMLST) method.

## 1. Introduction

Cardiovascular disease (CVD) is a prominent cause of death and mortality in the world, particularly in affluent countries [1,2]. The human gut microbiota contains over 1000 different gut microbiomes, which play significant roles in metabolism, minerals and vitamins bioactivation, immunological function, and digestion [3]. Food components such as choline, phosphatidylcholine, and L-carnitine can be metabolized by some gut bacteria and used as growth factors [4,5]. The gut microbiome and the extrinsic variables that cause disease have gotten much attention in recent years. The gut microbiota and its metabolites have been implicated in host physiology and metabolic disorders in numerous studies [3,6]. Trimethylamine-N-oxide (TMAO) is a chemical produced by gut microbial metabolism from choline, betaine, and carnitine. The host’s liver hepatic flavin-containing monooxygenase 3 (FMO 3) converts trimethylamine (TMA) to TMAO [7]. High plasma TMAO levels can be caused by gut dysbiosis. Studies have suggested that high serum TMAO levels have been linked to an increased risk of atherosclerosis [5], chronic kidney disease, cancer, and non-alcoholic fatty liver disease in the recent decade [5,8]. Therefore, lowering the serum TMAO level could be a potential target to prevent CVD and other diseases. Several therapeutic strategies have been investigated to target the reduction in TMAO levels, which includes the use of some analogs or the inhibition of TMA precursors. In these studies, serum TMAO levels were decreased by antibiotics and some natural or synthetic molecules such as allicin, 3,3-dimethyl-1-butanol (DMB), resveratrol (RSV), by remodeling the gut microbiota or inhibiting the activity of TMA lyase of TMA-producing bacteria [9]. Another proposed therapeutic approach is the use of probiotics potentially effective in altering the microbiota composition. Numerous studies suggested that the use of probiotics, single strain, or multistrain could influence the intestinal microbiome composition but failed to reduce serum TMAO levels [10,11,12,13].

The aim of this study is to find a strategy for the selection of potential probiotic strains that are able to reduce the TMAO level in vivo. In this study, we selected some lactobacilli with basic probiotic properties and used them for in vivo study in mice supplemented with choline. Additionally, to assure that our selected strains are unique strains, we also used genetic identification and phylogenetic methods to characterize our selected strains.

## 2. Materials and Methods

### 2.1. Bacterial Strains and Growth Conditions

Bacterial strains were collected from the Bioresource Collection and Research Center (BCRC, Hsinchu, Taiwan), the Deutsche Sammlung von Mikroorganismen und Zellkulturen (DSMZ, Germany) and the American Type Culture Collection (ATCC, Manassas, VA, USA). These bacteria stain numbers and sources, and growth conditions are shown in Table 1.

Stock cultures were maintained in 50% glycerol broth at −80 °C. Cells were grown at 37 °C for 18–24 h for DNA preparation, bacteria plate counting, and serial dilutions. Bacteria strains were incubated anaerobically at 37 °C using an anaerobic jar and anaeropack (MGC, Tokyo, Japan). Nutrient broth (NB) and tryptic soy broth (*TSB*) were purchased from Difco^TM^ (Becton, Dickinson and Company, Sparks, MD, USA) and *Acumedia* (Neogen, Lansing, MI, USA), respectively. The de Man, Rogosa, and Sharpe (MRS) agar medium was purchased from Difco^TM^ (USA). Agar *(Acumedia*, Neogen, Lansing, MI, USA) was mixed with NB/TSB/MRS at a final concentration of 1.5% (*w*/*v*). *Ten-*fold** serial *dilutions* of culture were prepared, 0.1 mL spotted on agar plates (*1.5%),* and *colony-forming units* per mL (CFU/mL) were counted.

### 2.2. Screening of Lactic Acid Bacteria (LAB) Strains and Their Antagonistic Effect against TMA-Producing Bacteria

#### 2.2.1. Resistance to Artificial Gastric and Intestinal Fluids

Simulated gastric digestion was tested essentially as described in Zarate et al. 2000 [14]. Simulated gastric juice prepared with the following composition: KCl 7 mmol L^−1^; NaCl, 125 mmol L^−1^; NaHCO3, 45 mmol L^−1^ and pepsin, 3 g L^−1^, HCl used to adjust the final pH 2 and 2.5 and with NaOH to pH 7. In general, 1 mL of cell suspension containing approximately 10^8^–10^9^ CFU mL^−1^ of LAB was transferred into 9 mL of simulated gastric juice with different pH 2, pH 2.5, and pH 7. The mixture was incubated at 37 °C for 0, 1.5, and 3 h. After incubation, viable LAB cells counts were determined by plating serial dilutions. Simulated intestinal fluid (SIF) was prepared using 0.1% (*wt*/*v*) pancreatin (Sigma) and 0.15% (*w*/*v*) Oxgall bile salts (Sigma) in water, and pH (pH 8.0) was adjusted with 5 mol l^−1^ NaOH. After 180 min of gastric digestion, cells were centrifuged (3000× *g*, 5 min), washed with PBS, suspended in SIF were incubated. Viable bacterial counts were taken at 0, 1.5, and 3 h by plating serial dilutions on MRS agar.

#### 2.2.2. Antimicrobial Activity of LAB Strains

The agar diffusion method was used to study the antibacterial activity of LAB strains. LAB strains were cultured individually in an MRS broth medium in anaerobic jars and incubated for 20 h at 37 °C. Bacterial cells were removed by centrifugation (5000× *g*, 5 min), and the supernatants were recovered. For each TMA bacteria strains, bacterial cells were cultivated on agar medium. Then, 100 μL of the spent culture supernatant (SCS) (10^8^ CFU/mL) of LAB was added, and the cultures were incubated at 37 °C for 14 h. The diameter of the inhibition zone around the wells was measured. LAB strains with inhibition zones < 11, 11–16, 17–22, and > 23 mm were classified as strains of no –; mild ++; strong ++; and very strong +++ inhibition, respectively [15].

#### 2.2.3. Adhesion to Caco-2 Cells for LAB Strains and TMA-Producing Strains

The conditions described by Gopal et al. [16] and Tsai et al. [17] for the adhesion study of LAB cells were followed. Caco-2 cells were maintained in Dulbecco’s Modified Eagle Medium (DMEM, Invitrogen, Basel, Switzerland) supplemented with 10% fetal bovine serum (FBS, Invitrogen) and 1% non-essential amino acids (NEAA, Invitrogen) at 37 °C in a humidified incubator (5% CO_2_). Caco-2 cells were transferred (5 × 10^4^ cells mL^−1^) to a 24-well multidish containing medium without antibiotics. The mixture was stored at 37 °C in a 5% CO_2_, 95% air atmosphere until a monolayer was formed in each well. Before the bacteria was added, the Caco-2 monolayer was washed twice with sterile PBS. The bacterial monocultures were grown overnight (18 h) and washed twice with sterile PBS. Bacteria cell suspension (1 × 10^8^ CFUmL^−1^) was added to a 24-well multidish and then incubated for 2 h. After incubation, it is washed three times with sterile PBS and fixed with formalin and stained with gram stain. The adhesion number of LAB cells was counted using Gopal et al. method [16]. The bacteria number was calculated in ten random microscopic fields.

### 2.3. Mouse Models

C57BL/6J female mice (5 weeks old) were purchased from National Laboratory Animal Center (NLAC), Taipei, Taiwan. The animal experiment was approved by the Institutional Animal Care and Use Committee (IACUC) of Hungkuang University (HK-10605). All mice were maintained at a controlled temperature of 23 ± 2 °C and relative humidity of 50–70% with a 12 h light/dark period. Each mouse was housed individually in cages and had free access to water and food *ad libitum*. All mice feed was purchased from Harlan Tekland (Madison, WI, USA). All mice were fed with a normal diet for 1-week adaptation (Table 2).

### 2.4. Probiotics Treatment

Lactobacilli, i.e., *Lpb. plantarum* LP1145, *L. amylovorus* LAM 1345 and *Lim. fermentum* LF33 were cultured individually in MRS broth at 37 °C. Bacterial strains were centrifuged and washed with PBS. Every day, 0.2 mL (10^9^ CFU/mL) of probiotic strains, i.e., multistrain formula (MF) or single strain, were given to each mouse in each treatment group (*n* = 7) by oral gavage until the end of the experiment. The probiotic mixture MF is the mixture of the selected strains in an equal volume of LAB solution, each containing 10^9^ CFU/mL. Blood samples were collected and allowed to clot at 4 °C, and serum samples were prepared by centrifugation (3000× *g* at 4 °C for 10 min). The serum aliquots were filtered and stored at −80 °C until analysis using UPLC-MS/MS.

### 2.5. UPLC-MS/MS Analysis

Derivatization of TMA and TMAO was carried out using ethyl bromoacetate according to the published methods with minor modifications [18,19,20,21,22,23,24,25,26,27,28,29,30]. Sample preparation and UPLC-MS/MS analysis conditions were previously published by Ramireddy et al. [19]. Briefly, 1 μL of samples were injected, and analysis was performed on Waters ACQUITY UPLC system with Xevo TQ MS (mass spectrometry), operated in positive ion electrospray ionization (ESI+) mode using MassLynx 4.1 SCN810 software. The precursor product ion pairs operated in multiple reaction monitoring (MRM) mode were: m/z146→118, m/z146.1→59 for TMA and d^9^TMA, m/z 76→59, m/z 76→42 for TMAO and d^9^TMAO, respectively.

### 2.6. Counting Lactobacilli Population in Cecum Digesta of Mice

On day 28, the cecum digesta taken from the mice of the MF group and LP1145 group after sacrifice was homogenized in PBS and centrifuged (1000× *g*). The upper phase was collected, followed by centrifugation. The pellet was then collected and used as intact bacterium cell suspension for DNA isolation using the EZNA stool DNA kit (Omega Bio-tek, Norcross, GA) following the manufacturer’s instructions. The extracted DNA samples were stored at −20 °C until use.

### 2.7. Quantification of *Lactobacilli* Populations by qPCR

To quantify the abundance of lactobacilli in mice intestines, real-time quantitative PCR (qPCR) was carried out using an ABI 7500 Fast system. Total DNA isolated from the mice cecum samples of control, MF, and LP1145 mice groups on day 28 after sacrifice was used for the detection of total lactobacilli counts. The Lactobacillus genus-specific primers were used, the primer sequences and PCR conditions are shown in Table 3. The final volume 20 μL of the real-time PCR mixture made up of 6.8 μL nuclease-free water, 10 μL SYBR premix, 0.4 μL ROX reference dye (KAPA Biosystems, Woburn, MA, USA), 2 μL of cDNA, and 0.4 μL (0.25 mM) each of the forward and reverse primers. For quantification of total lactobacilli, a standard curve was generated using a 10-fold dilution series standard ranging from 10^8^ to 10^2^ CFU/mL with real-time PCR. The bacterial cell count was calculated from a standard curve. All reactions were carried out in duplicate.

### 2.8. Identification of Lactobacillus Amylovorus Strain LAM 1345

To identify that strain LAM1345 is a unique strain, two tests were conducted, including biochemical method and genotypic identification. The preliminary tests were conducted for strain LAM1345, including gram staining, morphological observation, motility, catalase reaction, growth conditions (aerobic or anaerobic), whether endospores formed, and acid as well as bile tolerance. Gram staining and catalase by MRS agar (Difco lactobacilli MRS agar) were used to identify LAM1345 as LAB.

#### 2.8.1. Genotypic Characterization Method

##### DNA Extraction

Extraction of genomic DNA from the culture of LAM 1345 was performed using a Genomic DNA Mini Kit (Geneaid Biotech, GB100/GB300) according to the manufacturer’s protocol. The integrity, purity, and concentration of extracted DNA were confirmed absorbance at 260 nm by using a UV spectrophotometer (Eppendorf, Hamburg, Germany). The PCR primers and conditions are shown in Table 3. Each reaction amplification mixture (20 μL final volume) contained 10 ng of genomic DNA 1 μL, 10 μM of each primer 5 μL, 10 μL of 2 × Taq master mix (Ampliqon), dd water was added up to a 20 μL volume. The PCR product was analyzed by agarose gel electrophoresis (1.5% *w*/*v*) and visualized by staining with ethidium bromide. The PCR amplified product sent for 16S rDNA gene sequencing (Genomics BioSci & Tech Co., Ltd., Taipei Taiwan), obtained sequences were aligned using BLAST (http://www.ncbi.nlm.nih.gov/blast (accesses on 8 December 2020) [20]. Phylogenetic analysis was performed using clustalW [21].

#### 2.8.2. Leader Peptidase A (lep A) Gene Analysis

The LepA gene PCR primers and conditions are shown in Table 3. The PCR products were purified and sent to a sequencing company (Genomics Biosci and Tec Co. Taipei, Taiwan) to perform sequence analysis. The sequences were then compared with sequences of other strains using BLAST and clustalW.

#### 2.8.3. Phylogenetic Analysis

The neighbor-joining method was used to create a phylogenetic tree. To determine the stability of our phylogenetic tree, the percentage of replicate trees in which the associated taxa clustered together are shown next to the branches (1000 replicates) [22]. The Kimura 2-parameter method [23] was used to calculate the evolutionary distances. Codon positions 1st + 2nd + 3rd + noncoding were considered in the study, which included 17 nucleotide sequences. Gaps and missing data were removed from all positions. The total number of places in the final data set was 995. MEGA7 was used to perform evolutionary analysis.

### 2.9. Lpb. plantarum LP1145 Identification

#### 2.9.1. Genomic DNA Extraction, Sequencing, and Annotation

Genomic DNA for long reads using Oxford Nanopore Technologies (ONT) and short reads Illumina sequencing were extracted using QIAGEN Genomic-tip 20/G Kit. All extracted genomic DNA quality was determined using QuantiFluor^®^ dsDNA System. Sequencing was performed using ONT GridION for longer raw reads and Illumina Mini-Seq for short reads to generate a paired-end library of 2 × 310 bp for a large number of high accuracy short reads. ONT raw data obtained from the sequencer were decoded and demultiplexed on a sequencer by built-in software. The Illumina raw reads were quality trimmed (Phred Q score below 20) and de novo assembled using the CLC Genomics Workbench V.10., with default parameters. The validated ONT and Illumina sequences were assembled using the software SPAdes v3.13.0. Open reading frames (ORFs) were predicted using GLIMMER [24]. The rRNA and tRNA genes were annotated using RNAmmer and tRNAscan-SE, respectively. The identified coding regions were annotated by screening against the NCBI NT database using NCBI ncbi-blast 2.2.28+, while the translated sequences were searched against the NCBI NR database using DIAMOND with default parameters [25]. Average nucleotide identity (ANI) calculations were performed using the OrthoANIu tool EZBioCloud (https://www.ezbiocloud.net/tools/ani accessed on 11 December 2021) and digital DNA-DNA hybridization (dDDH) values were performed using the genome-to-genome distance calculation (GGDC) website (http://tygs.dsmz.de accessed on 11 December 2021). The ANI and dDDH values are shown in Table 4.

#### 2.9.2. Identification of Genes Related to Antimicrobial Resistance (AMR) and Virulence Factors, Biogenic Amine Producing Genes

BLASTp was used to find the AMR genes in the Comprehensive Antibiotic Resistance Database (CARD), NCBI AMRFinderPlus, ResFinder, and ARG-ANNOT (BLASTp, 70%coverage, 60% identity) [26]. To identify the virulence genes, BLAST searched against the Virulence Factors Database (VFDB), MvirDB, CGE Virulence Finder, CGE Pathogen Finder, and PAIDB [27]. The cutoffs were as follows: e-value (1.0e−20), coverage (70%) and identity (60%).

Microbial biogenic amine-related genes of tyrosine decarboxylase, histidine decarboxylase, ornithine decarboxylase, agmatine deiminase, and lysine decarboxylase were searched by BLASTn. The BLAST results showing a cutoffs e-value of 1.0e−20, identity (60%), and coverage >70% were considered.

#### 2.9.3. Whole-Genome Multilocus Sequence Typing (WgMLST)

The whole genome of *Lpb. plantarum* LP1145 was sequenced. The National Center for Biotechnology Information (NCBI) bacterial genome database was used to retrieve 51 public genome sequences of *Lpb. plantarum* strains (Table S1). The wgMLST analysis was performed using the cano-wgMLST _BacCompare web-based tool [28] as described by Huang et al. [29], to compare the genome sequences on the Linux platform.

The whole-genome sequences were compared with the constructed pan-genome allele database (PGAdb) using BLASTN V2.2.30 cano-wgMLST _BacCompare tool consists inbuilt process, Prokka pipeline, used for contig annotations, Roary V3.10.2 pipeline, for building PGAdb, PHYLIP v3.6 program [30] to construct a genetic relatedness tree.

### 2.10. Lim. fermentum LF1143 Identification

To confirm strain of LF1143 is a unique strain, in original *Lim. fermentum* sample (LF33), which was identified by the use of API 50 CHL kit, showed to be *Lim. fermentum* I and *Lim. fermentum* II, each counted about 42%–48%, respectively (data not shown). To identify the strain of LF1143, bacterial cells in the dd water-diluted solution of *Lim. fermentum* LF33 was cultured on MRS agar plate at 37 °C for 24 hrs.; then, single colonies were picked for further culture in MRS medium for bacteria cell collection as well as DNA preparation. The PCR conditions and primers specific for *Lim. fermentum* and *L. reuteri* strains are shown in Table 3.

### 2.11. Statistical Analysis

The findings were analyzed using a one-way analysis of variance (ANOVA) followed by Tukey’s multiple comparison test to establish statistical significance. Statistical significance was considered as *p* < 0.05. TMAO and TMA data from 7 mice in each experiment (control and probiotic groups) were taken for statistical analysis. Data were expressed as the mean ± standard deviation (SD) unless otherwise stated.

## 3. Results

### 3.1. Acid and Bile Tolerance among Lactobacilli

Based on the methods for the assay of the acid and bile resistance of the LAB strains, all the 6 LAB strains, i.e., *Lpb. plantarum* LP1145, *L. amylovorus* LAM1345, *Lim. fermentum* LF33 and other three strains, i.e., *L. gasseri* (BCRC 14619), *L. salivarius* (BCRC 14789), *Lpb. plantarum* (LPL07) were acid-tolerant at pH ≥ 2.5 and less tolerant at pH 2.0. In addition, LP 1145, LAM 1345, and LF33 strains are tolerant to bile. Only the strain of *L. gasseri* was less tolerant.

### 3.2. Growth Inhibition of TMA Bacteria by LAB Supernatants

Antimicrobial activity levels of LAB strains against TMA-producing bacteria are shown in Table 5. We randomly selected nine of the TMA bacteria strains and studied their inhibition by LAB strains. Probiotic strains used in this study were able to inhibit the growth of some of the TMA-producing bacteria, such as *Providencia alcalifaciens; Escherichia fergusonii; Proteus mirabilis; Klebsiella Pneumoniae; and Providencia rustigianii*, with inhibition zones greater than or equal to 15 mm. However, most of the LAB strains showed little or no antimicrobial activity against some of the TMA bacteria strains, such as *Clostridium sporogenes; Escherichia coli; Clostridium tetani*; and *Providencia rettgeri* strains, shown in Table 5.

### 3.3. Adherence of TMA Bacteria and Lactobacilli to Caco-2 Cells

We examined the adherence capacity of probiotic and TMA-producing strains to the human intestinal cell line Caco-2 (Table 6). All the strains examined were able to adhere to Caco-2 cells with variable levels. The adhered number of the TMA-producing strains showed poor adherence, i.e., less than 20 bacterial count per cell (Table 6). Probiotic strains showed variable ability to adhere to human Caco-2 cells, from 17.7 to > 200 CFU per Caco-2 cell. For strains of LAM 1345, LP1145, and *Lim. fermentum* LF33, which contains *Lim. fermentum* I and II as assayed with API 50 CHL kit, all these strains showed high adherence, i.e., > 200 CFU per Caco-2 cell. The probiotic strains exhibited stronger adherence to Caco-2 cells, and they may inhibit the adherence of TMA-producing strains by competition and exclusion.

### 3.4. Probiotic Strains Reducing Serum TMAO Levels in Mice Challenged with Choline

**Figure 1:**
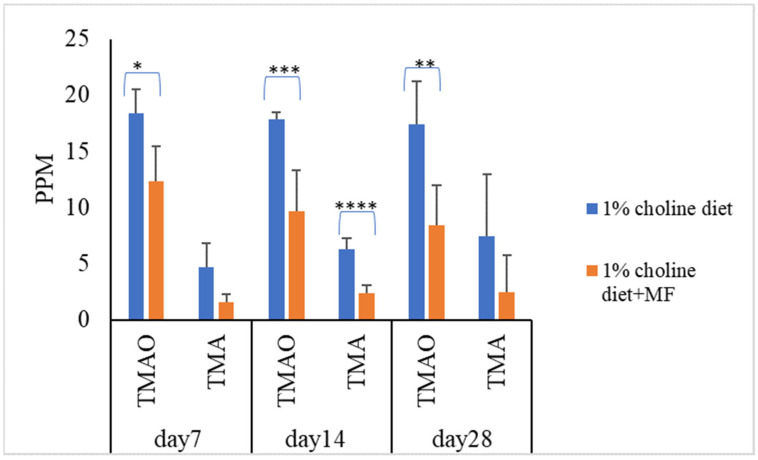
Probiotic multistrain formula decreases the plasma TMAO and TMA levels in C57BL/6J mice. Experimental conditions were as described in Methods. Data are shown as mean values ± SD (*n* = 7). A *p*-value of <0.05 was considered to be statistically significant, * *p* <0.05, ** *p* < 0.01, *** *p* <0.001, **** *p* < 0.0001.

To know the effect of probiotic strains on reducing serum TMAO levels in vivo, mice were divided into five groups (*n* = 7 in each experiment group). Mice in the control group were fed with a 1% choline diet, and mice in the other four groups were fed with 1% choline diet plus LAB multistrain formula (MF) or single LAB strains, i.e., LP1145, LAM 1345, LF1143 for 7, 14, and/or 28 days. After feeding, on day 7, day 14, and/or day 28, serum TMAO levels were measured. For MF group *Lim. fermentum* LF33 (contains type I and type II) and for single strain *Lim. fermentum* I -LF1143 was used for analysis. Results showed that probiotics in the MF group significantly reduced serum TMAO and TMA levels on days 7, 14, and 28 (Figure 1).

**Figure 2:**
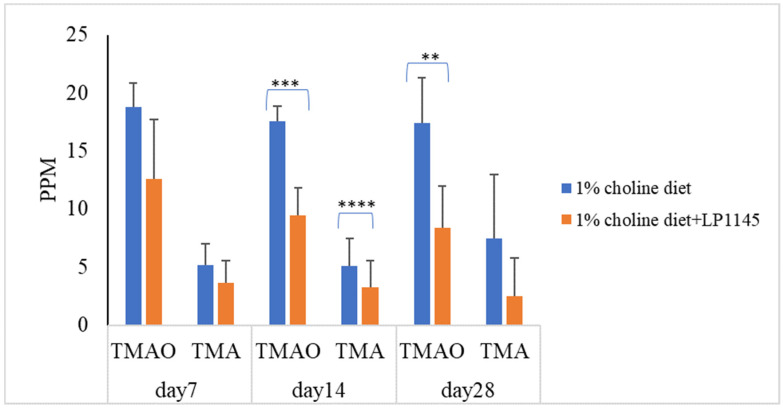
Probiotic strain *Lpb. plantarum* decreases the plasma TMAO and TMA levels in C57BL/6J mice. Experimental conditions were as described in Methods. Data are expressed as mean values ± SD (*n* = 7). ** *p* < 0.01, *** *p* <0.001, **** *p* < 0.0001.

Further, to find the roles played by the individual strains of LP1145, LAM 1345, and LF1143 on the serum levels of TMAO and TMA, attempts were made. The results showed that LP 1145 significantly reduced the serum level of TMAO on days 14 and 28 and TMA level on day 14 when compared with the result from the control group (Figure 2).

**Figure 3:**
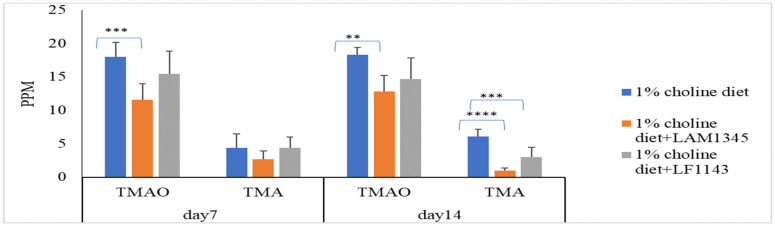
Effect of *L. amylovorusLAM1345* and *Lim. fermentum* LF1145 on serum TMAO and TMA levels. Experimental conditions were as described in Methods. Values are represented as mean values ± SD (*n* = 7) ** *p* < 0.01, *** *p* <0.001, **** *p* < 0.0001.

As for the effect of LAM1345 and LF1143 on TMAO and TMA levels in mice serum, LAM1345 showed a significant reduction effect on serum TMAO and TMA levels on day 7 and day 14 when compared to those from the control group (Figure 3). However, a significant reduction effect on serum TMAO levels was not observed for strain LF1143. Thus, the significant reduction in TMAO in mice serum by the MF group measured on days 7, 14, and 28 was mainly due to the contribution of strain LP1145 and LAM1345. In addition, for strains of LAM1345 and LF1143, since the results of that of TMAO reduction on day 14 were not improved as compared with that obtained on day 7, for these two strains, an animal study did not proceed to day 28.

**Figure 4:**
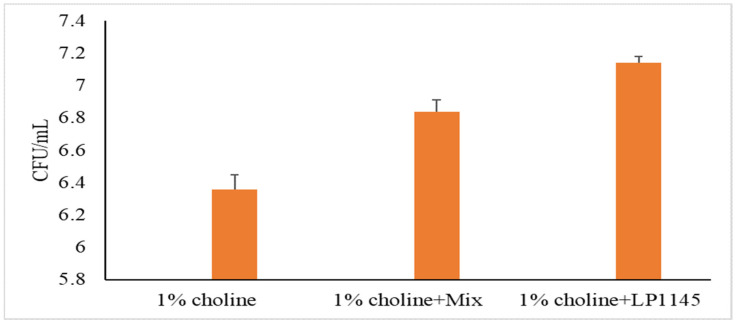
Total lactobacilli counts in mice cecum. Experimental conditions were as described in Methods. Values are shown in mean value ± SD (*n* = 7).

For MF and *Lpb. plantarum* groups, we also measured the intestinal total LAB counts after sacrificing the mice on day 28. A significant increase in lactobacilli population was observed as compared to that of the control group (Figure 4).

From the above study, we found two probiotic strains were able to reduce the TMAO and TMA level in the serum of mice challenged with choline., i.e., strain *L. amylovorus* LAM1345 and *Lpb. plantarum* LP1145. To assure that these strains are unique strains, we attempted to use molecular identification methods to distinguish these strains from other strains of the same species. In addition, to distinguish the strain of *Lim. fermentum* I from strain *Lim. fermentum* II, as described earlier, PCR primers specific for *Lim. fermentum* and *L. reuteri* were used to identify these strains.

### 3.5. Lactobacillus Amylovorus Identification

#### 3.5.1. Identification of Bacteria through Bacteriological and Biochemical Tests

The isolate was morphologically similar to *Lactobacillus* spp. and was found Gram-positive, catalase-negative, no motility, grown anaerobically, no endospore formation. The strain showed both acid and bile tolerance characteristics. The LAM 1345 strain is highly similar to *L. acidophilus*. If we use a biochemical method, such as API50, it may be detected as *L. acidophilus* strain. Thus, we performed genomic analysis to identify this strain.

#### 3.5.2. Sequence Analysis and Phylogenetic Tree

**Figure 5:**
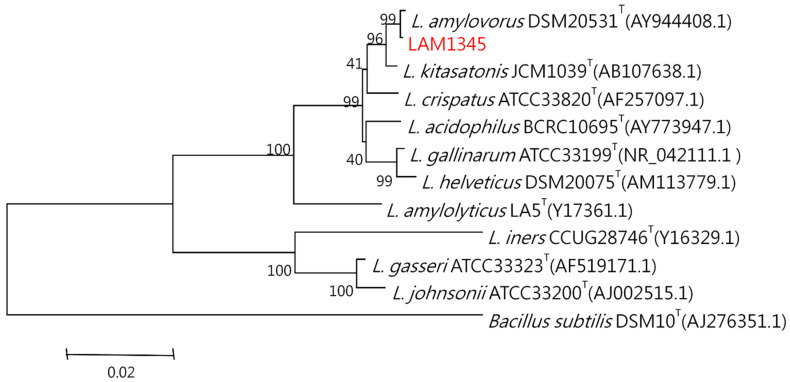
Phylogenetic tree based on the 16S rRNA gene sequence showing the phylogenetic relationships between the LAM1345 strain and related lactobacilli species.

**Figure 6:**
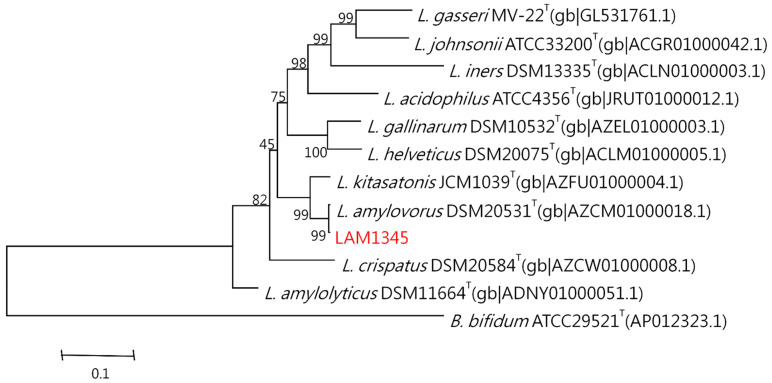
Phylogenetic tree of the lepA gene of strain LAM1345 strain and related lactobacilli species.

The genomic DNA was isolated according to the previously described method. The 16S rDNA was amplified at approximately 1500 bp, and the product was sequenced. In addition, the LepA gene of LAM1345 was amplified, and approximately 1163 bp of the DNA sequence was determined (unpublished data). DNA sequencing results were analyzed with NCBI-BLAST. MSA was analyzed using clustalW. The phylogenetic tree was depicted in Figure 5 and Figure 6, showing the genetic distances of various lactobacilli. The strain LAM 1345′ of 16s rDNA gene sequence was aligned with neighboring-type strains, and taxonomic relationships were determined. Highest degrees of 16s rDNA sequence identity with *L. amylovorus* DSM 20,531 ^T^ (accession number: AY944408.1, 99%) (Figure 5). Phylogenetic and homology analysis revealed that isolated strain LAM1345 could be *L. amylovorus*. Additionally, a phylogenetic tree to determine the relationship of the LepA gene inLAM1345. The strain LAM1345 showed the closest phylogenetic relationship with *L. amylovorus* DSM 20531 (accession number: AZCM01000018.1; 99%) (Figure 6).

### 3.6. Lpb. plantarum LP1145 Identification

The cano-wgMLST_BacCompare analysis platform was used to compare the genome sequences for strain typing of 52 *Lpb. plantarum* strains. The *Lpb. plantarum* PGAdb contained 10,273 genes, of which 2223 (21.6%) genes belonged to the core genome, 4713 (45.9%) belonged to the accessory genome, and 3337 (32.5%) were unique genes.

**Figure 7:**
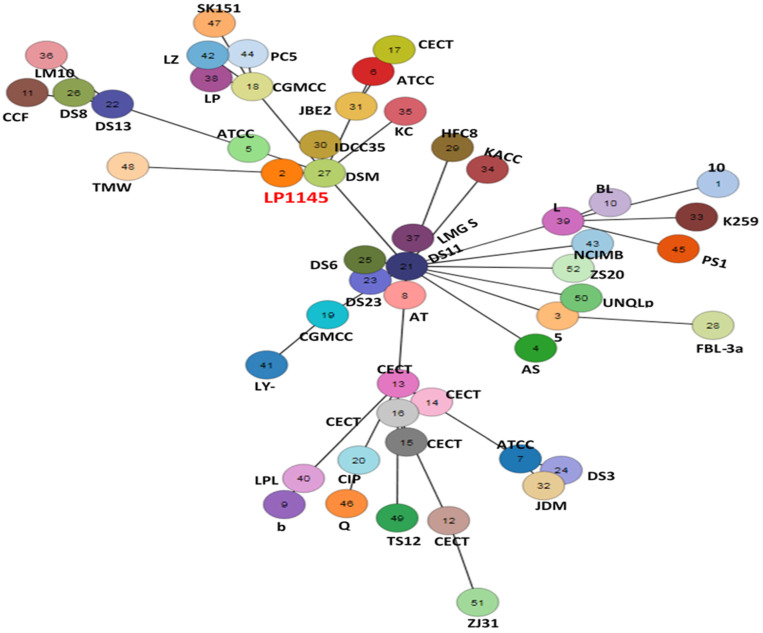
The allele-based minimum spanning tree constructed with cgMLST profiles for the 52 *Lactiplantibacillus plantarum* subsp. *plantarum* strains on the basis of a comparison of 2233 core genes. Each circle represents a different sequence type.

**Figure 8:**
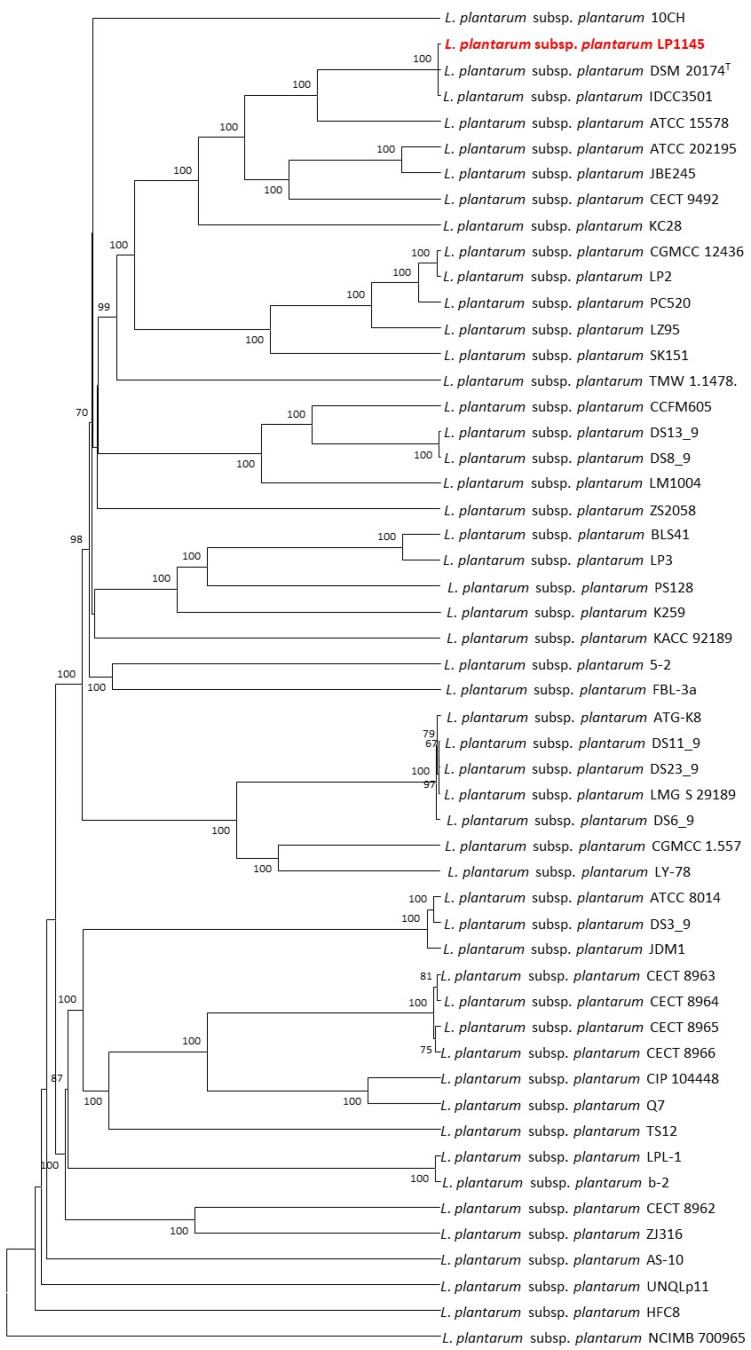
The allele-based UPGMA tree constructed with cgMLST profiles for the 52 *Lactiplantibacillus plantarum* subsp. *plantarum* strains on the basis of a comparison of 2233 differentiated core genes.

The 52 strains were assigned to different sequence types using the wgMLST analysis based on allele profiles of the 2223 core genes (Figure 7). The phylogenetic tree revealed that the strain LP1145 was most closely related to the DSM 20174^T^ (Figure 8) and showed 11 loci differences, including 35 SNPs, 3 insertion/deletions (Table S2).

### 3.7. Lim. fermentum Identification

To ensure that the strains we used were unique strains, we also tried to identify the strains in *Lim. fermentum* LF33 sample, if we use the API 50 CHL biochemical kit, two strains are identified as *Lim fermentum,* i.e., *Lim. fermentum* type I 48.5% and *Lim. fermentum* type II 42.8%. It was found that when we use specific PCR methods, it was found that in addition to strain LF1143, part of the strains are *L. reuteri* strains (initially *Lim. fermentum* II known as *L. reuteri*) (data not shown). We did not pursue molecular identification of these strains because serum TMAO levels were not considerably lowered by LF1143.

## 4. Discussion

Gut microbiota has received considerable attention because of its potential involvement in reducing the risk of cardiovascular disease (CVD) and other diseases. There is growing interest in research on the role of probiotics in the prevention and treatment of CVD and multiple diseases. The use of probiotics could be a safer and potentially more effective strategy to change the composition of the bacteria. A potential strain identification method is necessary for evaluating their probiotic properties. In this study, we found two novel LAB strains with potential probiotic properties and the ability to reduce the serum TMAO level in choline-challenged mice. According to the Food and Agriculture Organization and the World Health Organization (FAO/WHO), the requirement for probiotic strains are survival in the gastrointestinal tract, able to adhere to epithelial cells and cell lines, antimicrobial activity against potentially harmful microorganisms, ability to inhibit pathogen adherence to intestinal cell surfaces, and activity of the bile salt hydrolase [35]. In this study, the probiotic characteristics, including acid bile tolerance, antimicrobial activity, and adhesion capacity, were evaluated. The most significant properties of probiotics are their ability to survive in the stomach and intestine. The acid condition in the stomach and bile salt condition in the duodenum has been identified as the two most significant barriers to LAB survival in the host’s GI tract [36]. The probiotic strains used in this study showed enough tolerance to low pH and bile salts. Antimicrobial activity against TMA-producing bacteria was studied. These TMA production strains were able to produce TMA at different levels [19]. Most of the probiotic strains showed antimicrobial activity (Table 5). The capacity of probiotics to survive and adhere to the intestine is the key factor of probiotics. It not only allows probiotics to live longer in the GI tract but also enhances their interaction with the host. Probiotic strains we selected as potential strains have been found not only able to adhere to Caco-2 cells (Table 6) but also to stimulate the production of TNF-a by macrophage RAW264.7 (data not shown). Strain-specific cell-surface components play major roles in the adhesion ability of lactobacilli to the intestine mucosa. For *L. amylovorus* and *Lpb. plantarum* species, both have been shown able to increase their adherence to human epithelial cells due to their surface layer protein [37,38]. These surface layer proteins regulate the immune system by mediating bacterial adherence to the gut mucosa [39].

Probiotics are bacterial strains that lack the genes required to convert choline and carnitine to TMA [40]. Varied bacteria strains have different effects on choline metabolism and TMAO levels. Different bacteria strains can influence choline metabolism and TMAO levels in different ways. Recent studies have revealed that several bacteria strains such as Enterobacter aerogenes ZDY01, *Lactiplantibacillus plantarum subsp. plantarum* ZDY04, and *Lacticaseibacillus paracasei* can reduce TMAO and TMA levels by modifying the gut microbiota [41,42,43]. In contrast, in human clinical trials, supplementation of single or multistrain probiotics serum TMAO levels was not reduced [10,11,13,44,45]. New potential probiotic strains are needed to reduce TMAO levels in vivo and in humans. In this study, we have isolated new strains *L. amylovorus* LAM1345 and *Lpb. plantarum* LP1145 with probiotic properties. Reports have shown that *Lim. fermentum* and *L. amylovorus* are probiotics able to change body adiposity and gut microbiota in healthy people [28]. Our LAM1345, as well as LP1145, were able to reduce the TMAO level in serum and thus may be the potential probiotics able to lower the risk of CVD and other diseases.

The L. amylovorus strain has bile salt hydrolase (BSH) activity, which allows the deconjugation of bile salts; bile salt-activated signaling pathways, which are linked to metabolic disorders such as atherosclerosis, type 2 diabetes, obesity, and non-alcoholic steatohepatitis [46,47]. Study shows that daily consumption of *L. amylovorus* improves pre-obesity states and affects the gut microbial population [46]. However, no research was found on the effect of *L. amylovorus* on TMAO levels. This is the first study that we are aware of that focuses on TMAO level reduction by *L. amylovorus* strain.

Furthermore, we also use the high-resolution molecular methods to identify that our strain of LAM1345 is a novel strain. We tested physiological and biochemical properties. In general, rRNA and rDNA are the potential targets for the identification and phylogenetic analysis of these bacteria because of their ubiquity and their resistance to evolutionary changes. In this study, we sequenced 16srDNA and compared it with the NCBI database, and found that the identified strain belongs to *L. amylovorus*. Recently a unique elongation factor 4 (EF4), also known as LepA, was discovered in bacteria. LepA is one of the most conserved proteins found in all bacteria [48]. Sequencing and phylogenetic analysis showed that our isolate belongs to *L. amylovorus* with 99% similarity (Figure 6).

*Lpb. plantarum* plays an important role in the pharmaceutical industry and in the medical field without side effects [49]. A recent study showed *Lpb. plantarum* ZDY04 reduces the serum TMAO levels in mice [42]. However, how this strain can work in humans is unknown. In another study, supplementation of *Lpb. plantarum* 299v did not change the serum TMAO concentrations in men with coronary artery disease [45]. Finding a new potential probiotic strain to prevent CVD is necessary. In this study, we identified a new *Lpb. plantarum* LP1145 strain with probiotic properties, which significantly reduces the serum TMAO levels. Since *Lpb. Plantarum* ZDY04 has been published, although we do not have the ZDY04 strain for comparison. Comparative genomic analysis performed by the wgMLST method indicated that our LP1145 is a novel strain and closely related to the standard strain DSM20174^T^ (Figure 7 and Figure 8).

Our probiotic strain mixture, i.e., multistrain formula (MF), showed a significant reduction in serum TMAO levels from day 7 to day 28. For single strains, we selected *L. amylovorus*, *Lpb. Plantarum*, and *Lim. fermentum* type I (strain LF1143) rather than type II strain, i.e., *L. reuteri*, for effect on TMAO reduction study because all these three probiotic strains are effective in lowering the serum TMAO levels of mice. However, strain LF1143H may not be strong enough to reduce the serum TMAO level since *Lim. fermentum* has been shown to be a probiotic capable of modifying body adiposity and intestinal bacteria in healthy people. It may help to prevent CVD, cancer and diabetes, etc. [50]. These diseases have reported relatedness to high serum levels of TMAO. In addition, the multistrain formula has been reported to have additive and synergistic effects, and that may serve additional roles, such as improving strain colonization and adhesion [51]. Thus, the multistrain formula (MF), which contains the probiotic strains were able to reduce the serum level of TMAO. Finally, while all the investigations were carried out in mice, comparable outcomes could be expected in humans if the right conditions for human subjects and clinical trials are used. Currently, we are trying to use these LAB strains for a human clinical study.

## Figures and Tables

**Table 1:** List of bacterial strains used in the present study.

Strains	Strain No. ^a^	Medium ^b^	Source	Category
*Lactiplantibacillus plantarum*	LPL07	MRS	Lab isolate	Anaerobic
*Streptococcus thermophilus*	BCRC 13869;	TSB	Pasteurized milk	Aerobic
*Lactobacillus acidophilus*	BCRC 10695;	MRS	Human	Anaerobic
*Lactobacillus* *gasseri*	BCRC 14619;	MRS	Human mouth, gut, and feces	Anaerobic
*Ligilactobacillus salivarius*	BCRC 14759;	MRS	Saliva	Anaerobic
*Lactobacillus amylovorus*	LAM1345	MRS	Lab isolate	Anaerobic
*Limosilactobacillus fermentum*	LF1143	MRS	Lab isolate	Anaerobic
*Lactiplantibacillus plantarum*	LP1145	MRS	Lab isolate	Anaerobic
*Lactobacillus gasseri*	BCRC 17614;		Human	Anaerobic
*Lactobacillus gasseri*	BCRC 17615;	MRS	Human feces	Anaerobic
*Lactobacillus gasseri*	BCRC 17616;		Vaginal track	Anaerobic
*Ligilactobacillus salivarius*	BCRC 12574;	MRS	Saliva	Anaerobic
*Ligilactobacillus salivarius*	LSA1105	MRS	Lab isolate	Anaerobic
*Proteus mirabilis*	BCRC10725	NB	Urine of patient with kidney stones	Aerobic
*Klebsiella pneumoniae* subsp. pneumoniae	BCRC 10694	NB	Human urinary tract	Aerobic
*Escherichia fergusonii*	BCRC 15582	NB	Feces of 1-year-old boy	Aerobic
*Escherichia coli*	BCRC 15374	TSB	Human feces	Aerobic
*Clostridium sporogenes*	BCRC 10943	TSB	-	Anaerobic
*Clostridium tetani*	BCRC 80185	TSB	-	Anaerobic
*Providencia alcalifaciens*	BCRC 13995	NB	Human feces	Aerobic
*Providencia rettgeri*	BCRC 12624	NB	-	Aerobic
*Providencia rustigianii*	BCRC 13997	NB	Human feces	Aerobic
*Anaerococcus hydrogenalis*	BCRC 80846	TSB	Human feces	Anaerobic
*Anaerococcus vaginalis*	BCRC 80848	TSB	Ovarian abscess	Anaerobic
*Anaerococcus tetradius*	BCRC 80847	TSB	Human vagina	Anaerobic
*Hungatella hathewayi*	BCRC 80852	TSB	Human feces	Anaerobic
*Yokenella regensburgi*	BCRC 80857	NB	Human wrist wound	Aerobic
*Vibrio furnissii*	BCRC 80856	NB	Human feces	Aerobic
*Olsenella uli*	BCRC 80854	TSB	Human gingival crevice	Anaerobic
*Klebsiella pneumoniae* subsp. rhinoscleromatis	BCRC 17593	NB	Nose of a patient, Sumatra	Aerobic
*Klebsiella variicola*	BCRC 80853	NB	-	Aerobic
*Klebsiella oxytoca*	BCRC 17136	NB	-	Aerobic
*Proteus penneri*	BCRC 80855	NB	Urine	Aerobic

^a^ ATCC: American Type Culture Collection (Virginia); DSM: Deutsche Sammlung von Mikroorganismen und Zellkulturen (Germany); BCRC: Bioresource Collection and Research Center (Taiwan) ^b^ deMan Rogosa Sharpe (MRS); nutrient broth (NB); tryptone soya broth (TSB).

**Table 2:** Choline-deficient diet. After the adaptation period, mice were fed either a choline-deficient diet or a normal diet with 1% choline chloride. The experiment was carried for 2–4 weeks, and during the experimental period, the blood of the mice was sampled from the eyes on 0, 7, 14, and/or 28 days.

	g/kg
Casein, “vitamin-free” test	193.0
DL-methionine	3.0
Sucrose	508.4543
Corn starch	151.2497
Corn oil	50.0
Cellulose	50.0
Mineral mix, AIN-76(170915)	35.0
Calcium carbonate	4.0
Vitamin E, DL-alpha tocopheryl acetate (500 IU/g)	0.242
Vitamin A palmitate (500,000 IU/g)	0.0396
Vitamin D3, cholecalciferol (500,000 IU/g)	0.0044
Vitamin mix, w/o choline, A, D, E (83171)	5.0
Ethoxyquin, antioxidant	0.01

**Table 3:** Primers and PCR conditions used in this study.

	Primer Sequences	No. of PCR Cycles and PCR Conditions	Size (bp)	Ref
Lactobacillus	F: 5′-GACTTGGTTGAAATGGAAGT-3′ R: 5′-TCAGTGGTGTGGAAGTAGAA-3′	40 (35 s at 95 °C, 35 s at 54 °C, 35 s at 72 °C) 40 s at 72 °C	572	[31]
16 s	F:5′AGAGTTTGATCCTGGCTCAG-3′ R: 5′-GGTTACCTTGTTACGACT-3′	30 (60 s at 94 °C, 60 s at 50 °C, 60 s at 72 °C) 7 min at 72 °C	1500	[32]
lepA	F: 5′-GGDCACGTRGAYTTYTCWTAYGA-3′ R:5′-GCATAVCCYTTNGTDRAWGACTT-3′	35 (60 s at 94 °C, 75 s at 50 °C, 60 s at 72 °C) 7 min at 72 °C	1163	In this study
*Lim. fermentum*	F: 5′-AATACCGCATTA CAACTTTG -3′ R: 5′-GGTTAAATACCCTCAACGTA -3′	35 (60 s at 94 °C, 60 s at 50 °C, 60 s at 72 °C) 7 min at 72 °C	337	[33]
*L. reuteri*	F: 5′-CAGACAATCTTTGATTGTTTAG-3′ R: 5′-GCTTGTTGGTTTGGGCTCTTC-3′	40 (30 s at 95 °C, 60 s at 60 °C, 120 s at 2 °C) 2 min at 72 °C	300	[34]

**Table 4:** Pairwise comparison of average nucleotide identity (ANI) and digital DNA-DNA (dDDH) hybridization values between *Lpb. plantarum* and the closely related type strains.

Query Strain	Subject Strain	dDDH (%)	ANI (%)
LP1145	*Lactiplantibacillus plantarum* ATCC 14917	100	99.99
LP1145	*Lactiplantibacillus plantarum* DSM 20174	100	99.97
LP1145	*Lactobacillus arizonensis* DSM 13273	94.1	99.23
LP1145	*Lactiplantibacillus argentoratensis* DSM 16365	62.9	95.64
LP1145	*Lactiplantibacillus paraplantarum* DSM 10667	31.1	86.22

**Table 5:** Inhibition of TMA-producing bacteria by lactic acid bacteria.

	*Lpb.* *plantarum* LP1145	*Streptococcus thermophilus* BCRC 13869	*L. amylovorus* LAM1345	*L. gasseri* BCRC14619	*L. salivarius* BCRC14759	*L.* *acidophilus* BCRC 10695	*Lim.* *fermentum* LF1143	*Lpb. plantarum* LPL07	*L. gasseri* BCRC17614	*L. gasseri* BCRC 17615	*L. gasseri* BCRC 17616	*L. salivarius* BCRC 12574	*L. salivarius* LSA1105
*Providencia alcalifaciens*	21 [++]	21 [++]	24 [+++]	22 [++]	27 [+++]	20 [++]	25.5 [+++]	22 [++]	19.5 [++]	20.5 [++]	20.5 [++]	25 [+++]	27.5 [+++]
*Escherichia* *fergusonii*	23.5 [+++]	18.5 [++]	19.5 [++]	20.5 [++]	17.5 [++]	22.5 [+++]	21 [++]	23 [+++]	17 [++]	19 [++]	17 [++]	17.5 [++]	18 [++]
*Proteus mirabilis*	24 [+++]	20.5 [++]	21.5 [++]	21 [++]	19 [++]	24 [+++]	20.5 [++]	22 [++]	17 [++]	18.5 [++]	18 [++]	19 [++]	19 [++]
*Klebsiella Pneumoniae*	21.5 [++]	18 [++]	19.5 [++]	19 [++]	17.5 [++]	21 [++]	19 [++]	20.5 [++]	17 [++]	17.5 [++]	17.5 [++]	17.5 [++]	18 [++]
*Providencia rustigianii*	27 [+++]	26 [+++]	21.5 [++]	22 [++]	23 [+++]	26.5 [+++]	26 [+++]	24.5 [+++]	22 [++]	22.5 [+++]	23.5 [+++]	24 [+++]	23.5 [+++]
*Clostridium sporogenes*	11 [−]	13 [+]	9 [−]	12.5 [+]	13.5 [+]	9 [−]	9 [−]	11 [−]	12 [+]	22 [++]	13.5 [+]	12.5 [+]	13 [+]
*Escherichia coli*	12 [+]	11.5 [+]	12 [+]	12 [+]	12 [+]	10 [−]	11 [−]	11 [−]	12.5 [+]	11 [−]	10 [−]	11 [−]	11.5 [+]
*Clostridium tetani*	9 [−]	9 [−]	10 [−]	11 [−]	13 [+]	9 [−]	9 [−]	9 [−]	11 [−]	11.5 [+]	12 [+]	14 [+]	13 [+]
*Providencia rettgeri*	14 [+]	11 [−]	11.5 [+]	12 [+]	11.5 [+]	12 [+]	12 [+]	12 [+]	12 [+]	11 [−]	11 [−]	11 [−]	11.5 [+]

Control group MRS broth 100 uL, inhibition zone 9 mm; lactic acid bacteria count 10^9^; LAB strains with inhibition zones ≤ 11, 11–16, 17–22 and > 23 mm were classified strains of no [−]; mild [+]; strong [++] and very strong [+++] inhibition, respectively.

**Table 6:** Adhesion of TMA and LAB bacteria to Caco2 cells.

Strains	TMA and LAB Bacteria per Caco2 Cells (mean ± SD)
Negative control	0 ±0
*Escherichia coli*	19.2 ± 4.8
*Providencia rettgeri*	3.4 ± 1.3
*Providencia alcalifaciens*	4.6 ± 3.2
*Providencia rustigianii*	5.5 ± 2.1
*Clostridium sporogenes*	5.8 ± 2.0
*Clostridium terani*	9.9 ± 4.1
*Escherichia fergusoni*	11.2 ± 2.8
*Proteus mirabilis*	13.5 ± 6.5
*Klebsiella pneumonia*	12.0 ± 4.7
*Klebsiella sub rhinoscleromatis*	6.7 ± 2.9
*Klebsiella varicola*	14.6 ± 5.3
*Klebsiella oxytoca*	13.1 ± 2.1
*Anaerococcus hydrogenalis*	5.5 ± 2.0
*Anaerococcus vaginalis*	7.2 ± 2.2
*Anaerococcus tetradius*	6.0 ± 1.8
*Hungatella hathewayi*	7.4 ± 3.3
*Yokenella regensburgi*	10.6 ± 2.5
*Proteus penneri*	17.8 ± 3.9
*Vibrio furnisii*	12.7 ± 3.2
*Olsenella uli*	15.1 ± 5.3
*Lactobacillusgasseri* (14619)	23.5 ± 7.8
*Ligilactobacillus salivarius* (14789)	25.1 ± 3.5
*Lactiplantibacillus plantarum* LPL07	18.7 ± 3.2
*Lactobacillus amylovorus* LAM1345	273 ± 1.32
*Limosilactobacillus fermentum* LF1143	218 ± 2.36
*Lactiplantibacillus plantarum* LP1145	203 ± 2.37

## Data Availability

Not applicable.

## Supplementary Materials

The following are available online at https://www.mdpi.com/article/10.3390/foods10122931/s1, Table S1: Genomic characteristics of *Lactiplantibacillus plantarum subsp. plantarum* strains. Table S2: Discriminated loci between *Lactiplantibacillus plantarum subsp. plantarum* LP1145 and DSM 20174^T^.
