# Supplementary material for: Molecular Identification and Selection of Probiotic Strains Able to Reduce the Serum TMAO Level in Mice Challenged with Choline
Source: Foods. 2021 Nov 27;10(12):2931. doi: 10.3390/foods10122931 (PMC8700464; doi:10.3390/foods10122931)
Supplement: Supplementary file 1 [file foods-10-02931-s001.zip › foods-1426812-supplementary.pdf]

**Table S1.** Genomic characteristics of *Lactobacillus plantarum* strains.

| Strain no. | Source | Accession no. | Genome size (bp) | G+C content (%) | No. of contigs | No. of CDS |
|---|---|---|---|---|---|---|
| 10CH | Cheese | GCA_002005385.2 | 3,311,056 | 44.5 | 1 | 3,079 |
| AS-10 | Fruits and vegetables | GCA_003045645.1 | 3,022,969 | 44.8 | 1 | 2,853 |
| ATCC 15578 | Brewery | GCA_002900075.1 | 3,473,609 | 44.1 | 27 | 3,319 |
| ATCC 202195 | Human | GCA_004354995.1 | 3,327,225 | 44.3 | 20 | 3,139 |
| ATCC 8014 | — | GCA_002749655.1 | 3,309,473 | 44.5 | 5 | 3,109 |
| ATG-K8 | Kimchi | GCA_003597615.1 | 3,275,764 | 44.5 | 2 | 3,066 |
| BLS41 | Kimchi | GCA_002116955.1 | 3,476,011 | 44.2 | 6 | 3,289 |
| b-2 | Pickle | GCA_003352125.1 | 3,211,268 | 44.8 | 1 | 3,039 |
| CCFM605 | Yogurt-like dairy product | GCA_003344825.1 | 3,314,628 | 44.4 | 55 | 3,167 |
| CGMCC 1.557 | — | GCA_001272315.2 | 3,273,239 | 44.4 | 3 | 3,104 |
| CGMCC 12436 | Yogurt-like dairy product | GCA_003344845.1 | 3,259,936 | 44.5 | 53 | 3,046 |
| CIP 104448 | Human stool | GCA_000956195.1 | 3,237,330 | 44.4 | 178 | 3,043 |
| CECT 8962 | — | GCA_900289155.1 | 3,290,117 | 44.3 | 136 | 3,106 |
| CECT 8963 | — | GCA_900290135.1 | 3,248,428 | 44.4 | 136 | 3,062 |
| CECT 8964 | — | GCA_900290105.1 | 3,247,322 | 44.4 | 173 | 3,033 |
| CECT 8965 | — | GCA_900290125.1 | 3,342,151 | 44.3 | 152 | 3,144 |
| CECT 8966 | — | GCA_900290085.1 | 3,295,692 | 44.3 | 161 | 3,093 |
| CECT 9492 | Chenopodium quinoa seeds | GCA_900695295.1 | 3,343,723 | 44.2 | 63 | 3,153 |
| DS11_9 | Commercial dietary supplements | GCA_003061805.1 | 3,208,332 | 44.5 | 25 | 2,998 |
| DS13_9 | Commercial dietary supplements | GCA_003053185.1 | 3,112,570 | 44.7 | 32 | 2,913 |
| DS23_9 | Commercial dietary supplements | GCA_003053045.1 | 3,204,801 | 44.5 | 38 | 3,991 |
| DS3_9 | Commercial dietary supplements | GCA_003053165.1 | 3,231,607 | 44.5 | 36 | 3,027 |
| DS6_9 | Commercial dietary supplements | GCA_003061725.1 | 3,215,836 | 44.5 | 71 | 3,001 |
| DS8_9 | Commercial dietary supplements | GCA_003053025.1 | 3,113,711 | 44.7 | 27 | 2,910 |
| DSM 20174$^T$ | Pickled cabbage | NZ_CP039121.1 | 3,250,154 | 44.5 | 2 | 3,032 |
| FBL-3a | Beef cattle | GCA_003999275.1 | 3,232,240 | 44.6 | 1 | 3,045 |
| HFC8 | Faecal | GCA_001302645.1 | 3,405,709 | 44.3 | 11 | 3,370 |
| IDCC3501 | Kimchi | GCA_003428355.1 | 3,242,652 | 44.5 | 1 | 3,022 |
| JBE245 | Meju | GCA_001596095.1 | 3,262,611 | 44.5 | 1 | 3,056 |
| JDM1 | — | GCA_000023085.1 | 3,197,759 | 44.7 | 1 | 2,985 |
| KACC 92189 | — | GCA_003692595.1 | 3,460,294 | 44.3 | 5 | 3,270 |
| KC28 | Kimchi | GCA_002948215.1 | 3,291,849 | 44.5 | 1 | 3,118 |
| K259 | Kimchi | GCA_002868775.1 | 3,373,076 | 44.5 | 2 | 3,175 |
| LM1004 | Kimchi | GCA_002895245.1 | 3,198,690 | 44.6 | 1 | 3,006 |
| LMG S 29189 | — | GCA_002914965.1 | 3,200,023 | 44.5 | 19 | 2,996 |
| LP1145 | Fermented vegetables | This study | 3,250,380 | 44.5 | 2 | 3,032 |
| LP2 | Pickles | GCA_002109425.1 | 3,284,622 | 44.5 | 1 | 3,082 |
| LP3 | Commercial dietary supplements | GCA_002286275.1 | 3,329,954 | 44.4 | 3 | 3,103 |
| LPL-1 | Fermented fish | GCA_002205775.2 | 3,200,572 | 44.6 | 2 | 3,006 |
| LY-78 | Fermented chinese cabbage | GCA_001715615.1 | 3,128,783 | 44.6 | 2 | 2,926 |
| LZ95 | Baby stool | GCA_001484005.1 | 3,322,458 | 44.5 | 3 | 3,126 |
| NCIMB 700965 | Cheese | GCA_003611015.1 | 3,216,285 | 44.5 | 6 | 3,207 |
| PC520 | Chinese fermented food-pickles | GCA_002576835.1 | 3,452,904 | 44.3 | 3 | 3,286 |
| PS128 | Fu-tsai | GCA_001005805.1 | 3,325,806 | 44.6 | 11 | 3,087 |
| Q7 | Yak fermented milk | GCA_003999605.1 | 2,981,934 | 44.6 | 5 | 2,809 |
| SK151 | Kimchi | GCA_003269405.1 | 3,231,249 | 44.6 | 1 | 2,997 |
| TMW 1.1478 | Honey | GCA_003345375.1 | 3,387,168 | 44.3 | 2 | 3,198 |

| | | | | | | |
|---|---|---|---|---|---|---|
| TS12 | Stinky Tofu | GCA_001908455.1 | 3,433,628 | 44.3 | 7 | 3,379 |
| UNQLp11 | Pinot noir wine | GCA_004730965.1 | 3,534,932 | 44.2 | 1 | 3,413 |
| ZJ316 | Healthy newborn fecal sample | GCA_000338115.2 | 3,299,755 | 44.5 | 4 | 3,138 |
| ZS2058 | sauerkraut | GCA_001296095.1 | 3,198,337 | 44.7 | 1 | 2,958 |
| 5-2 | Fermented soybean | GCA_001278015.1 | 3,237,652 | 44.7 | 1 | 3,034 |

**Table S2.** Discriminated loci between *Lactobacillus plantarum* LP1145 and DSM 20174$^{T.}$

| Locus no. | Gene | Annotation | Length (bp) | Position (SNP) | Insertion/Deletion (nucleotide sequence) |
|---|---|---|---|---|---|
| | — | IS1182 family transposase[*] | 1655 | 11(G/A), 87(C/A), 1473(A/T), 1477(T/C), 1590(G/A), 1597(C/A), 1621(G/A) | Ins (C) |
| 2 | — | major facilitator superfamily transporter | 1455 | 278(C/T) | |
| 3 | — | IS1182 family transposase[*] | 1851 | 478(A/G) | |
| 4 | — | sigma-54-dependent transcriptional regulator | 2850 | 2444(A/C) | |
| 5 | — | IS1182 family transposase[*] | 1701 | 224(G/A), 498(C/T), 982(A/G), 1196(G/A), 1309(A/G) | |
| 6 | guaA | Glutamine -hydrolyzing GMP synthase | 1557 | 889(T/G) | |
| | — | MarR family transcriptional regulator | 477 | 264(T/G) | |
| 7 | — | Cna B-type domain-containing protein | 2958 | | Del (TGCTACTGCTAC) |
| 8 | ccpA_2 | Catabolite control protein A | 1011 | 954(A/C) | |
| 9 | — | NAD(P)/FAD-dependent oxidoreductase | 1203 | 805(C/A) | |
| 10 | — | gamma-D-glutamyl-meso-diaminopimelate peptidase | 789 | 339(T/C) | |
| 11 | — | DUF5011 domain-containing protein | 3347 | 218(C/T), 290(T/C), 314(T/C), 401(T/C), 425(C/T), 2340(G/A), 2488(A/G), 2492(T/C), 2559(T/C), 2590(G/A), 2690(T/C), 2728(A/G), 2820(A/G), 3311(C/T), 3312(A/C) | Del (AACCAGTAGTG-CAGCGAACAGTGT) |

[*]multicopy gene.
